# Regulation of Thrombin-Induced Lung Endothelial Cell Barrier Disruption by Protein Kinase C Delta

**DOI:** 10.1371/journal.pone.0158865

**Published:** 2016-07-21

**Authors:** Lishi Xie, Eddie T. Chiang, Xiaomin Wu, Gabriel T. Kelly, Prasad Kanteti, Patrick A. Singleton, Sara M. Camp, Tingting Zhou, Steven M. Dudek, Viswanathan Natarajan, Ting Wang, Steven M. Black, Joe G. N. Garcia, Jeffrey R. Jacobson

**Affiliations:** 1 Institute for Personalized Respiratory Medicine, University of Illinois at Chicago, Chicago, Illinois, United States of America; 2 Department of Medicine, University of Illinois at Chicago, Chicago, Illinois, United States of America; 3 Department of Medicine and Arizona Respiratory Center, University of Arizona, Tucson, Arizona, United States of America; 4 Department of Medicine, University of Chicago, Chicago, Illinois, United States of America; 5 Department of Pharmacology, University of Illinois at Chicago, Chicago, Illinois, United States of America; Section of Pulmonary and Critical Care Medicine, UNITED STATES

## Abstract

Protein Kinase C (PKC) plays a significant role in thrombin-induced loss of endothelial cell (EC) barrier integrity; however, the existence of more than 10 isozymes of PKC and tissue–specific isoform expression has limited our understanding of this important second messenger in vascular homeostasis. In this study, we show that PKCδ isoform promotes thrombin-induced loss of human pulmonary artery EC barrier integrity, findings substantiated by PKCδ inhibitory studies (rottlerin), dominant negative PKCδ construct and PKCδ silencing (siRNA). In addition, we identified PKCδ as a signaling mediator upstream of both thrombin-induced MLC phosphorylation and Rho GTPase activation affecting stress fiber formation, cell contraction and loss of EC barrier integrity. Our inhibitor-based studies indicate that thrombin-induced PKCδ activation exerts a positive feedback on Rho GTPase activation and contributes to Rac1 GTPase inhibition. Moreover, PKD (or PKCμ) and CPI-17, two known PKCδ targets, were found to be activated by PKCδ in EC and served as modulators of cytoskeleton rearrangement. These studies clarify the role of PKCδ in EC cytoskeleton regulation, and highlight PKCδ as a therapeutic target in inflammatory lung disorders, characterized by the loss of barrier integrity, such as acute lung injury and sepsis.

## Introduction

The lung endothelium serves as a dynamic, semi-permeable barrier between the interstitial alveolar spaces and the circulating blood, with the integrity of this cellular barrier vital to pulmonary homeostasis. Endothelial cell (EC) barrier function is highly regulated by incompletely defined stimulus–coupling pathways. We have previously shown that protein kinase C (PKC), a Ser/Thr kinase plays an important role in the regulation of EC barrier function [1–3]. Earlier studies have shown that activators of PKC such as phorbol esters and diacylglycerol increase the permeability of the endothelial monolayer, whereas PKC inhibitors reduce the cell barrier disruption caused by thrombin, bradykinin, platelet derived growth factor (PDGF), hydrogen peroxide and vascular endothelial growth factor (VEGF) on neutrophils [4]. A full understanding of the contribution of PKC to EC barrier function is limited as PKC is a superfamily that comprises at least 10 members whose expression and functions are differentially regulated [5]. Each cell type expresses a particular combination of PKC isozymes that elicit diverse and sometimes opposing responses [6], thereby making it difficult to infer the exact role of PKC in physiological and pathological conditions.

Studies based on the use of isoform-specific inhibitors, peptides, or antisense oligonucleotides have identified PKCα and PKCβ as important signaling mediators in the regulation of vascular permeability [4]. Further, it was reported that PKCδ, but not PKCα, β1, or ε, is required in phorbol ester-induced EC barrier disruption [7]. Activation of PKCδ is also critical for maintenance of basal barrier function, which is correlated with enhanced focal adhesion formation, actin filament stabilization, and RhoA activation [8–10]. Interestingly, it was reported that hyperglycemia induced the upregulation of PKCδ dependent signaling in pericytes leading to increased endothelial permeability and pathologic progression of diabetic retinopathy [11].

In the present study, we identified differential expression of several PKC isoforms in EC. In response to thrombin, human pulmonary artery EC revealed a strong translocation of PKCδ from the cytosol to the membrane, a hallmark of activation and functionality of the kinase [5, 12]. In addition, studies using a chemical activator of PKCδ revealed dose-dependent barrier disruption. Moreover, thrombin-induced barrier disruption was partially attenuated by inhibiting PKCδ activation by pretreating the cells with rottlerin or by expressing a dominant negative form of PKCδ. Our findings indicate that PKCδ partly mediates thrombin-induced activation of RhoA and myosin light chain (MLC) phosphorylation and the subsequent barrier disruption. Protein kinase D (PKD) and CPI-17 are well known PKCδ targets [7, 13–15]. Our inhibitory studies suggest that these targets are downstream of thrombin-induced PKCδ activation in lung EC. In addition, thrombin appears to suppress Rac1 activation that is both PKCδ dependent and independent. Taken together, these studies suggest that PKCδ inhibition may provide a therapeutic strategy to block loss of endothelium integrity in inflammatory lung disorders.

## Materials and Methods

### Reagents

Human thrombin (cell culture grade), fetal bovine serum (FBS), phosphate buffer saline, and bovine serum albumin were purchased from Sigma-Aldrich (St. Louis, MO). Phorbol-12-myristate-13-acetate (PMA), and rottlerin were from EMD/Calbiochem (La Jolla, CA). Anti-PKC (isoform specific), anti-beta-actin and anti-phospho-CPI-17 (Thr38) antibodies were obtained from Santa Cruz Biotech (Santa Cruz, CA); and anti-diphospho-MLC (Thr18/Ser19), anti-total MLC antibodies and anti-phospho-PKD (Ser916) antibodies were from Cell Signaling Technology (Beverly, MA). Anti-mouse and anti-rabbit secondary antibodies conjugated to horse radish peroxidase, enhanced chemiluminescence (ECL), and ECL-Plus were purchased from Amersham Biosciences, Inc /GE Health Sciences (Piscataway, NJ). Texas Red-phalloidin, and Prolong mounting solution were from Molecular Probes (Eugene, OR).

### Cell Culture

Human pulmonary artery EC were purchased from Lonza Group, Ltd (Switzerland). Endothelial Growth Medium-2 was prepared with defined growth factors supplemented up to 10% FBS. Cells were grown at 37°C in a 5% CO_2_ incubator and used from passage 6–9. For these experiments, EC were plated at appropriate density and used 3 d later unless otherwise stated.

### Subcellular Fractionation

The procedure is based on ultracentrifugation steps as previously described [11]. Briefly, cells were lysed on 10 cm plates using 500 μl of buffer A (20 mM Tris-HCl, pH 7.5, 2 mM EDTA, 0.5 mM EGTA, 1 mM phenylmethylsulfonyl fluoride, 25 μg of leupeptin per ml, 0.1 mg of aprotinin per ml, 0.33 M sucrose) and centrifuged at 1000 x g for 10 min to eliminate cell debris; and the supernatant was then subjected to ultracentrifugation at 100,000 x g for 30 min at 4°C. The supernatant was retained as the cytosolic fraction. The membrane fraction pellets were resuspended in buffer B (buffer A without sucrose) with 1% TritonX-100. After incubating for 30 min, soluble membrane fractions were obtained by ultracentrifugation at 100,000 x g for 30 min.

### Immunofluorescence

EC were seeded in an 8-chamber culture dish with collagen-coated Culture Slides (BD Biosciences, Lexington, KY). Cells were washed with PBS and fixed with 3.7% formaldehyde for 5 min, permeabilized with 0.25% Triton X-100 in PBS for 3 min, washed again and probed with Texas Red-phalloidin at 1:200 dilution. Slides were then mounted with Prolong™ anti-fade reagent. Stained cells were visualized using a Nikon Eclipse TE2000 inverted microscope (Nikon Inc., Melville, NY).

### Electrical Resistance Measurements

Electrical cell-substrate impedance sensing (ECIS) system (Applied Biophysics, Troy, NY) was used to measure transendothelial electrical resistance (TER) with EC grown on gold microelectrodes [16–18]. EC were plated directly onto gold microelectrodes of an ECIS plate (8W10E) and cultured for 2–3 d. Confluency was assessed as minimum basal resistance of 1400 ohms. Data pooling and analysis were performed using Epool software [19].

### Rac1 and RhoA Activation Assays

The activation levels of Rac1 and RhoA were measured using commercially available kits (Millipore, Bedford, MA). Briefly, cell lysates were collected after specified treatment, and GTP-bound Rac1 or RhoA were captured using pull-down assays with PAK1-PBD or Rhotekin-RBD beads according to the manufacturer’s protocol. The captured proteins were then subjected to immunoblotting to determine the levels of active GTP-bound Rac1 or RhoA using specific antibodies.

### Western Blotting

Cells were washed with cold Endothelial Basal Medium (EBM) once and solubilized in 0.3% SDS lysis buffer (300 μl/D60) containing protease inhibitors (1 mM EDTA, 1 mM PMSF, 1 mM sodium orthovanadate, 1 mM sodium fluoride, 0.2 TIU/ml aprotinin, 10 μM leupeptin, 5 μM pepstatin A). Genomic DNA was sheared with a 26-gauge syringe. Each sample was boiled for 5 min, and diluted with 5X sample buffer (0.56 M Tris pH 7.0, 10% SDS, 25% β-ME, 25% sucrose, and 0.025% bromophenol blue). Sample proteins were separated in 10% or 15% SDS-PAGE gel using the Mini-Protean II (Bio-Rad, Hercules, CA) and then transferred onto Immobilion-P PVDF membranes (Millipore, Bedford, MA). Immunoblotting was performed with primary antibodies (1:1000, 4°C overnight) followed by secondary antibodies conjugated to HRP (1:5000, 30 min) and detected with ECL or ECL-Plus on Biomax MR film (Kodak, Rochester, NY). Integrated densities of bands were quantified using ImageJ software (Research Services Branch, Bethesda, MD).

### Adenovirus infection

Control LacZ and dominant-negative PKCδ over-expressing adenoviruses, provided by Dr. Motoi Ohba (Showa University, Japan) [20], were generated and validated at the Gene Transfer Vector Core at the University of Iowa. EC were plated accordingly, and the media was replaced with serum-free media at half volume containing the adenoviral construct (MOI = 25–100 pfu/cell). After 1 h of serum-free infection, an equal volume of growth medium was supplemented and the entire medium was replaced 24 h post-infection. Cells were used after 72 h post-transfection and viral infection efficiency was confirmed by either measuring β-gal activity or PKC expression in cell lysates.

### Transfection of small interfering RNA (siRNA)

The siRNA specific for PKCδ was commercially obtained (Dharmacom, Lafayette, CO). Human pulmonary EC were transfected with siRNA using siPORT™Amine (Invitrogen, CA) according to the manufacturer’s instruction. Briefly, after 1h transfection with 100 nM siRNA in serum-free medium, an equal volume of growth medium was added and the entire medium replaced 24 h later. Cells were used for biochemical or functional assays 72h post-transfection.

### Endothelial permeability transwell assay

EC were seeded at 4×10^4^ cells/well in a final volume of 100 μl growth medium, the inserts (Cat. #3470, Coning, NY) were placed into 24-well plates containing 400 μl medium overnight. To measure thrombin-induced EC permeability changes, 100 μl FITC-dextran (1 mg/ml, M. W = 50 kD) was added into the insert and incubated for 1 h. The insert was then removed and 100 μl medium was collected from the bottom chamber. The fluorescent intensity of FITC-dextran in the samples was determined using CYTO FLUOR multi-well plate reader Senes 4000 (Perspective Biosystems, CA).

### Statistical analysis

Student’s test was used to compare means of two or more different treatment groups. The level of significance was set to p< 0.05 unless otherwise stated. Data are expressed as Mean± SE.

## Results

### Thrombin induces PKCδ activation in human lung EC

**Fig 1:**
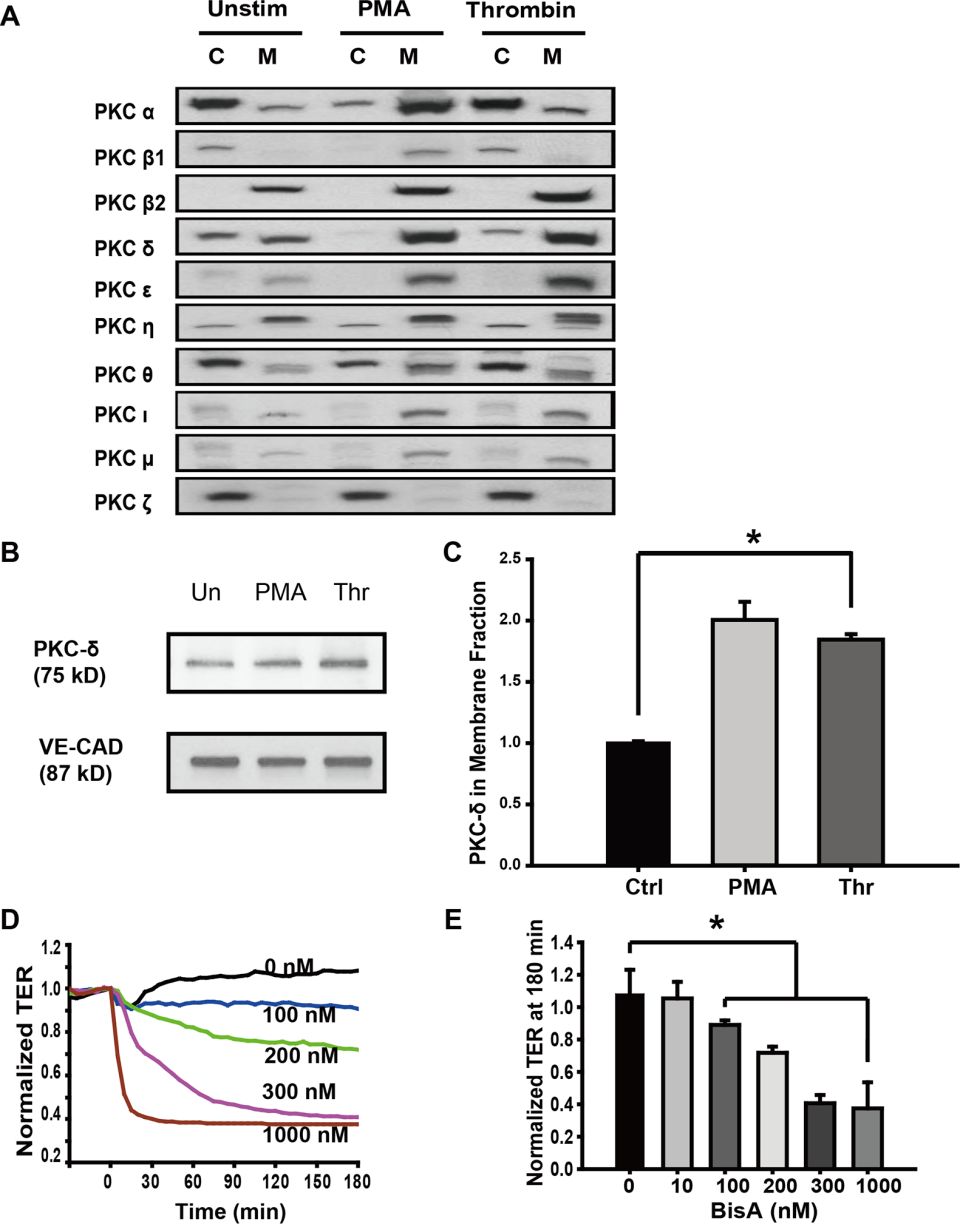
The role of PKCδ in thrombin-induced endothelial barrier disruption. **(A)** Cells were treated with thrombin (1 U/ml, 15 min) or PMA (100 nM, 30 min) with unstimulated cells (Unstim) used as controls. Cytosolic (C) and membrane (M) fractions were immunoblotted with various anti-PKC isoform-specific antibodies. Both thrombin and PMA increased PKCδ in the membrane fraction and correspondingly decreased it in the cytosol fraction thereby indicating that PKCδ is activated and translocated to membranes. (**B**) Cells were treated as Fig 1A, Membrane fractions were immunoblotted with PKCδ and VE-Cadherin specific antibodies. (**C**) Normalized densitometry of PKCδ in membrane fraction is shown (n ≥ 3/condition, * p < 0.05). (**D)** Human pulmonary artery EC were grown to full confluency on gold microelectrodes and then treated with 100–1000 nM BisA, a putative activator of PKCδ. BisA induced a dose-dependent decrease in TER corresponding to increased permeability. Representative traces are shown. (**E)** TER values in BisA-treated cells at 180 min are shown (n = 4/condition, * p < 0.05 compared to control).

To analyze the participation of PKC isoforms in EC barrier regulation, human pulmonary artery EC were treated with thrombin, an edemagenic agent, and the activation status of 10 PKC isoforms were determined based on their translocation from the cytosol to the membrane (a marker for activation although specific isoforms localize to the membrane without activation). As shown in Fig 1A, compared with the untreated group, PMA, a well-known PKC activator, resulted in the translocation of most of the PKC isozymes, while thrombin induced significant accumulation of PKCβ2, δ, ε, ι, and μ; in the membrane compartment. Untreated cells revealed the presence of significant amounts of the PKCδ enzyme in both cytosolic and membrane fractions and in response to thrombin, there was a preferential accumulation of the PKCδ isoform in the membrane fraction as shown in Fig 1B and 1C, decreased V-Cad was presented as positive control for Thrombin-induced effect. There was no change in the relative levels of PKCθ and PKCζ between thrombin-treated and untreated cells.

### PKCδ activation contributes to thrombin-induced barrier disruption

**Fig 2:**
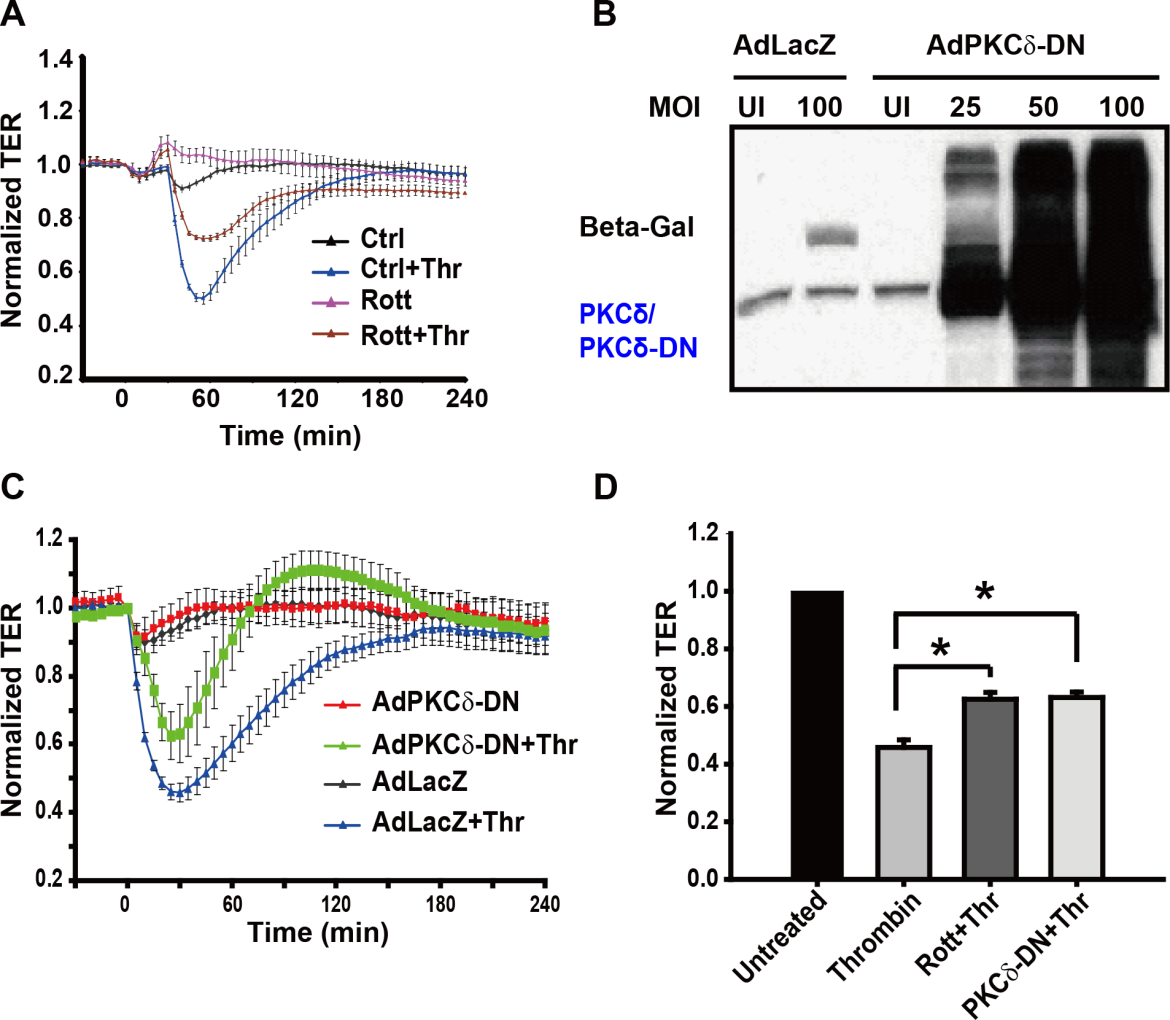
Effects of rottlerin pretreatment and expression of dominant negative PKCδ on thrombin-induced endothelial barrier dysfunction. **(A)** EC monolayer leakiness was assessed by measuring TER in EC pretreated with rottlerin (10 μM, 30 min), a PKCδ inhibitor, followed by thrombin stimulation (1 U/ml, 15 min). Error bars = ± SE. (**B)** EC were infected with either control adenovirus (AdLacZ) or Ad PKCδ-DN (MOI: multiplicity of infection). After 3 d, cells were lysed and immunoblotted. **(C)** Infected EC (MOI = 25) were then challenged with thrombin (1 U/ml) and TER (normalized to basal resistance) measured over time. Error bars = ± SE. (**D)** Maximum decrease in TER (normalized to basal resistance) in EC treated with either rottlerin or infected with Ad PKCδ-DN is shown. (n = 6/condition, * p < 0.05).

Previous studies by Harrington *et al*., using rat lung and epididymal microvascular EC reported a basal barrier integrity enhancing function for PKCδ. Given the heterogeneity of vascular EC in humans [9], we asked whether a similar function could be attributed to PKCδ in human EC. We used putative activator and inhibitor of PKCδ in our studies to determine the role of PKCδ in the regulation of EC barrier integrity. Bistratene A, a putative PKCδ activator [21], stimulates PKC delta translocated from cytosol to membrane [22]. As shown in Fig 1D and 1E, the effect of Bistratene A, was an increase in EC permeability over a period of 3 h in a dose-dependent manner as measured by TER. These data suggest that PKCδ activation promotes the loss of barrier integrity in human lung EC. Conversely, thrombin-induced decreases in TER were partly attenuated when pretreated with rottlerin, a putative inhibitor of PKCδ [23] (Fig 2A). To rule-out possible non-specific effects of rottlerin, we suppressed PKCδ signaling by overexpression of a dominant negative form of PKCδ (PKCδ-DN) in EC. Consistent with rottlerin pretreatment, overexpression of PKCδ-DN had a similar attenuating effect on thrombin-induced barrier disruption (Fig 2B and 2C). As shown in the bar graph of normalized TER values at 30 min (Fig 2D), treatment with either rottlerin or overexpression of PKCδ-DN significantly ameliorated thrombin-induced EC barrier disruption. Taken together, these data indicated a modest but significant protective effect of PKCδ inhibition on EC barrier integrity upon thrombin exposure.

### PKCδ inhibition attenuates thrombin-induced actin stress fiber formation

**Fig 3:**
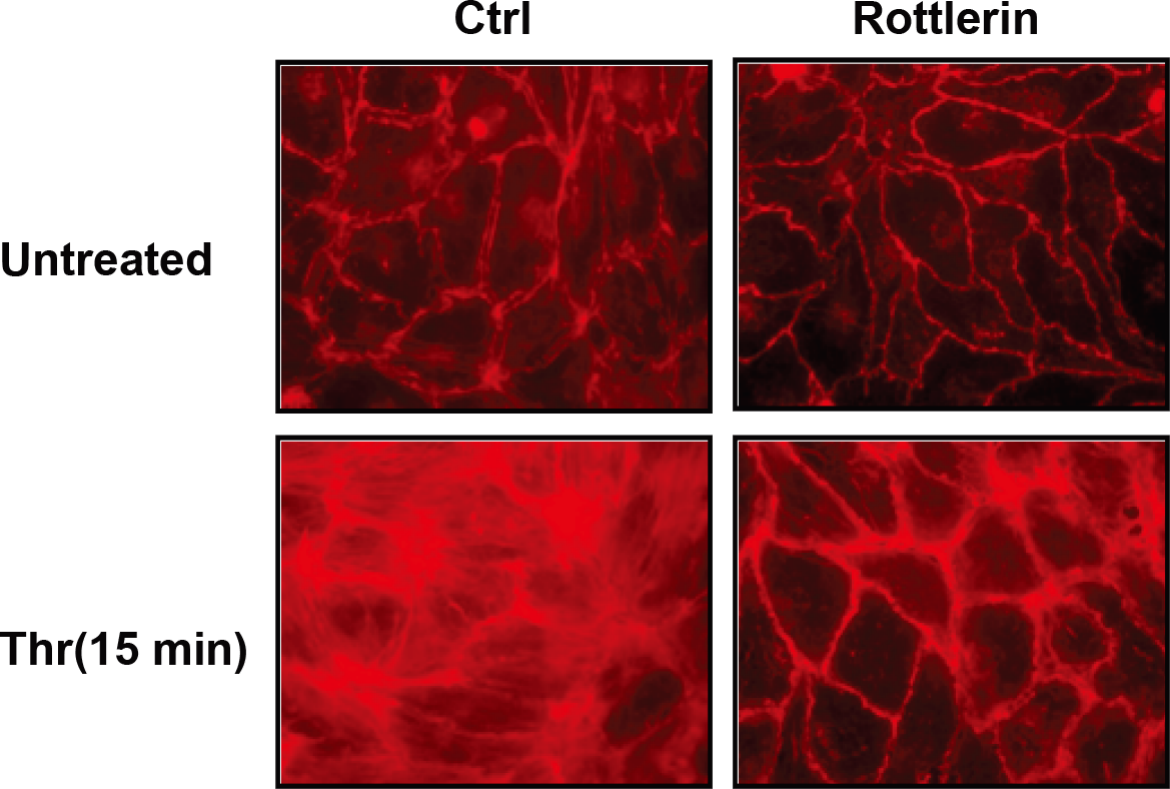
Inhibition of PKCδ abrogates thrombin-induced stress fiber formation. Untreated (top left panel), rottlerin pretreated (10 μ;M, 30 min; top right panel), thrombin treated (bottom left panel), and rottlerin pretreated EC exposed to thrombin (bottom right panel) were fixed, permeabilized, stained for F-actin with Texas-Red phalloidin and imaged by fluorescence microscopy (representative images shown).

As thrombin-induced barrier disruption is characterized by enhanced EC actin stress fiber formation [24] we next assessed the effect of rottlerin on these events. Pretreatment with rottlerin induced a sharper delineation of the peripheral actin ring in comparison to untreated controls. In addition, thrombin induced prominent central actin stress fiber formation that was abrogated by rottlerin pretreatment (Fig 3). These results suggest a role for PKCδ in modulating EC barrier integrity through the regulation of actin stress fiber formation.

### PKCδ mediates thrombin-induced MLC phosphorylation

**Fig 4:**
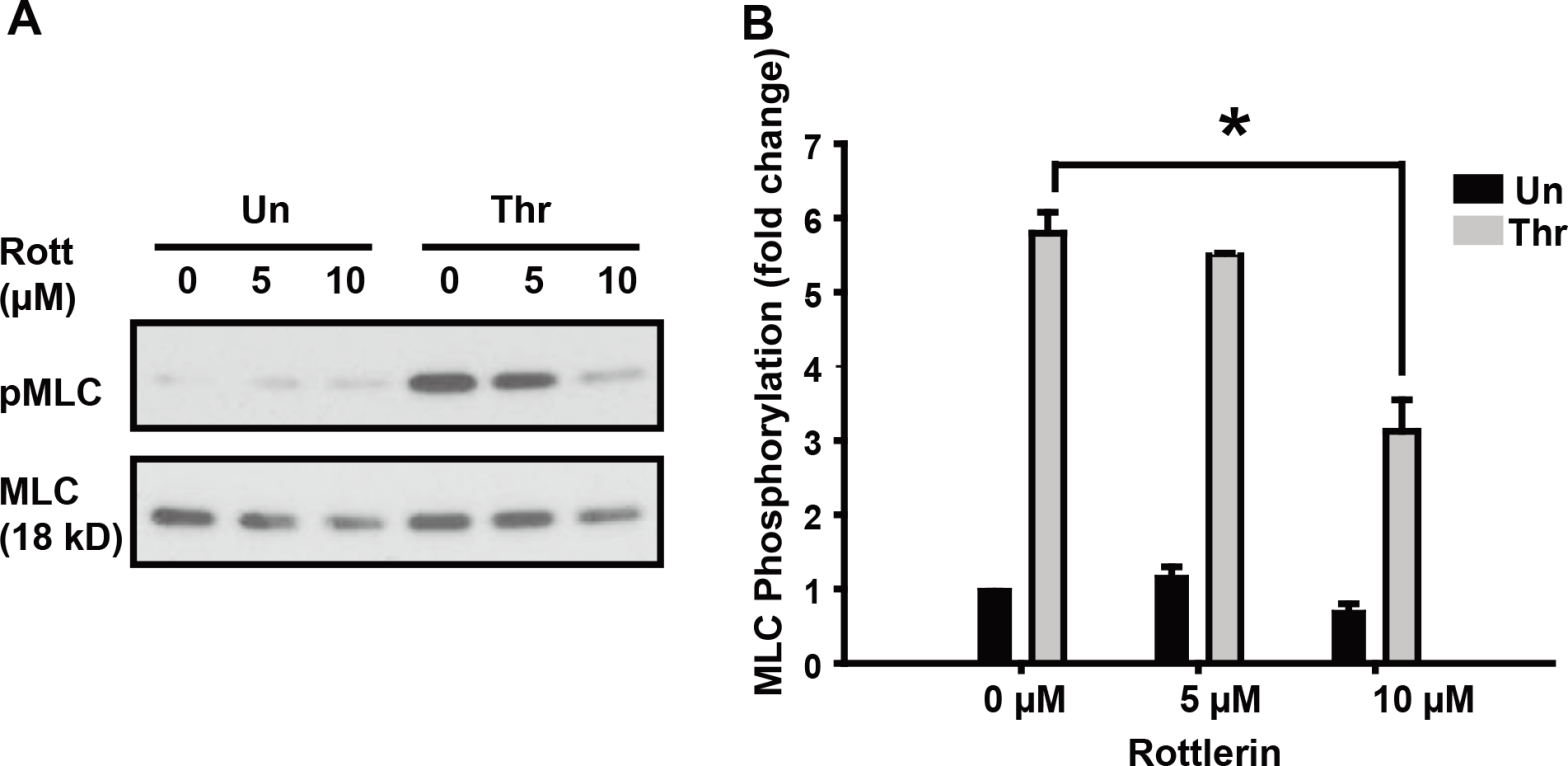
Effect of PKCδ inhibition on thrombin-induced MLC phosphorylation. (**A)** EC were pretreated with rottlerin (5–10 μM, 30 min) and subsequently stimulated with thrombin (1U/ml, 5 min). Whole cell lysates were immunoblotted with anti-phospho-MLC (Thr18/Ser19) antibody. (**B)** Relative MLC phosphorylation by densitometry is shown (n ≥ 3/condition, * p < 0.05).

The involvement of PKCδ in growth factor-induced MLC activation has been reported in various cell types [25–27]. To analyze the involvement of PKCδ in thrombin-induced MLC activation, we pretreated EC with rottlerin, a PKC inhibitor. As shown in Fig 4A and 4B, rottlerin pretreatment attenuated thrombin-induced MLC phosphorylation in a dose-dependent manner, suggesting that thrombin-induced EC MLC phosphorylation requires the activation of PKCδ.

### PKCδ regulates Rho GTPases activity upon thrombin exposure

**Fig 5:**
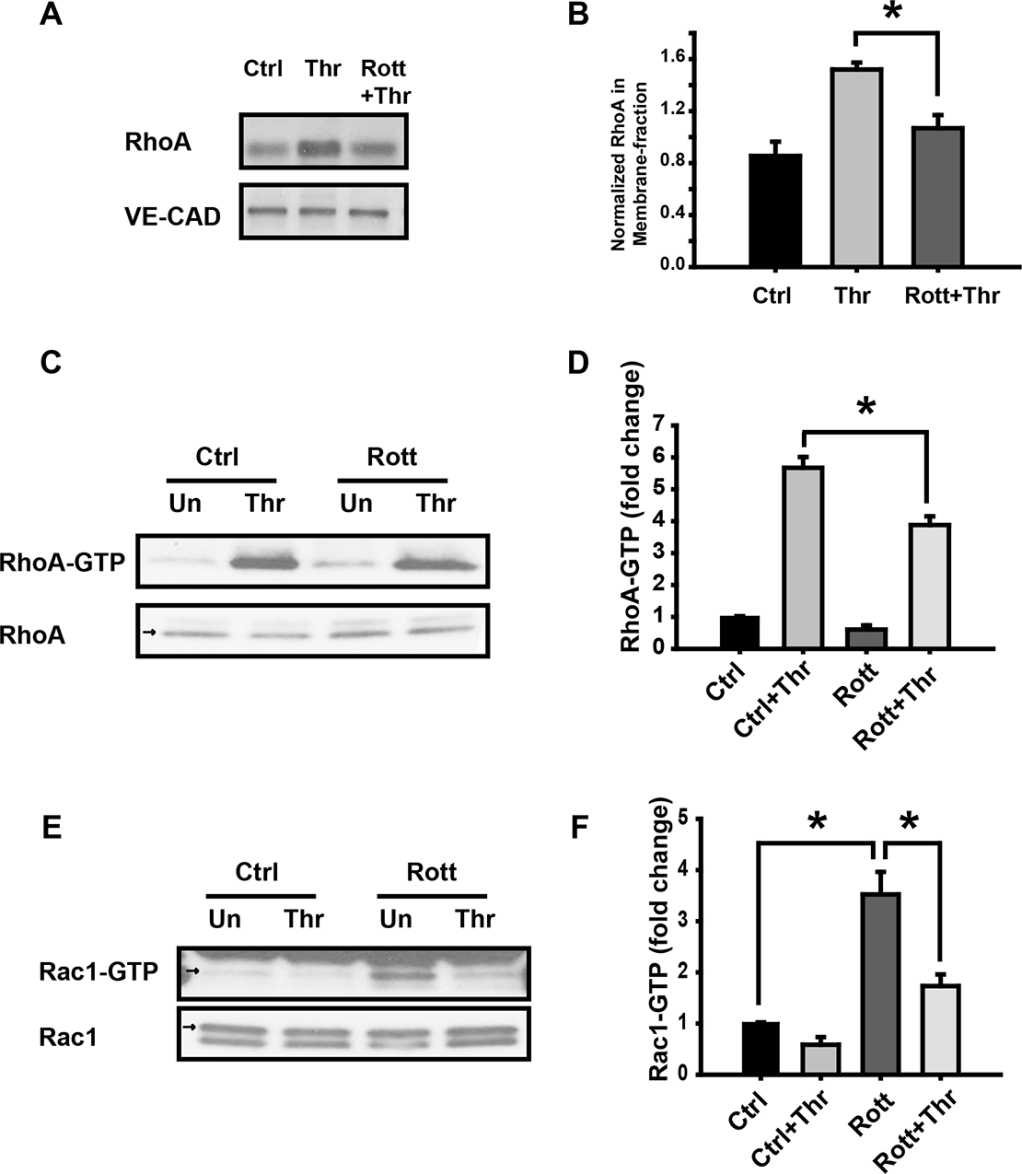
Effect of PKCδ inhibition on thrombin-induced changes in Rho and Rac GTPase activity. EC were pretreated with rottlerin (10 μM, 30 min) followed by exposure to thrombin (1 U/ml, 5 min). Unstimulated cells (Un) were used as controls. (**A)** Membrane fractions were immunoblotted with RhoA and VE-Cadherin specific antibodies. (**B)** Normalized densitometry of RhoA in membrane fraction is shown. (**C)** Activated RhoA (RhoA-GTP) was determined as described in the methods. **(D)** Normalized RhoA activity based on densitometric analysis is shown. (**E)** Using the same experimental conditions, Rac1 activation (Rac1-GTP) was determined as described in the methods. (**F)** Normalized Rac1 activity based on densitometric analysis is shown. n≥ 3/condition, * p < 0.05.

Rho GTPases play a critical role in regulating EC permeability [28]. It is well established that thrombin-induced stress fiber formation is mediated by the RhoA/ROCK pathway [29]. To assess whether the activities of thrombin-induced Rho GTPases are affected by PKCδ, we measured the amount of GTP-bound RhoA and Rac1 (active forms) in relation to their total amounts under conditions of rottlerin pretreatment followed by thrombin challenge in EC [30–32]. As shown in Fig 5A and 5B, rottlerin-pretreatment attenuated thrombin-induced RhoA membrane translocation, which is in agreement with the RhoA-GTP data shown in Fig 5C and 5D, where there was a dramatic activation of RhoA in thrombin-treated cells that was only partially suppressed by rottlerin-pretreatment. These data suggest that PKCδ is upstream of RhoA activation and that thrombin induces RhoA activation through more than one signaling pathway. In contrast to RhoA, thrombin had no discernible effect on Rac1 activation (Fig 5E and 5F). Pretreatment with rottlerin, however, resulted in a dramatic increase in Rac1 activity that was partly suppressed in thrombin-treated cells, suggesting that PKCδ has a suppressive effect on Rac1 activity. Importantly, thrombin appears to further suppress Rac1 activity even in rottlerin pretreated cells thereby suggesting that PKCδ independent mechanisms also significantly contribute to the observed thrombin-mediated suppression of Rac1 activation.

### PKD and CPI-17 are potential targets of PKCδ in thrombin-induced signaling

**Fig 6:**
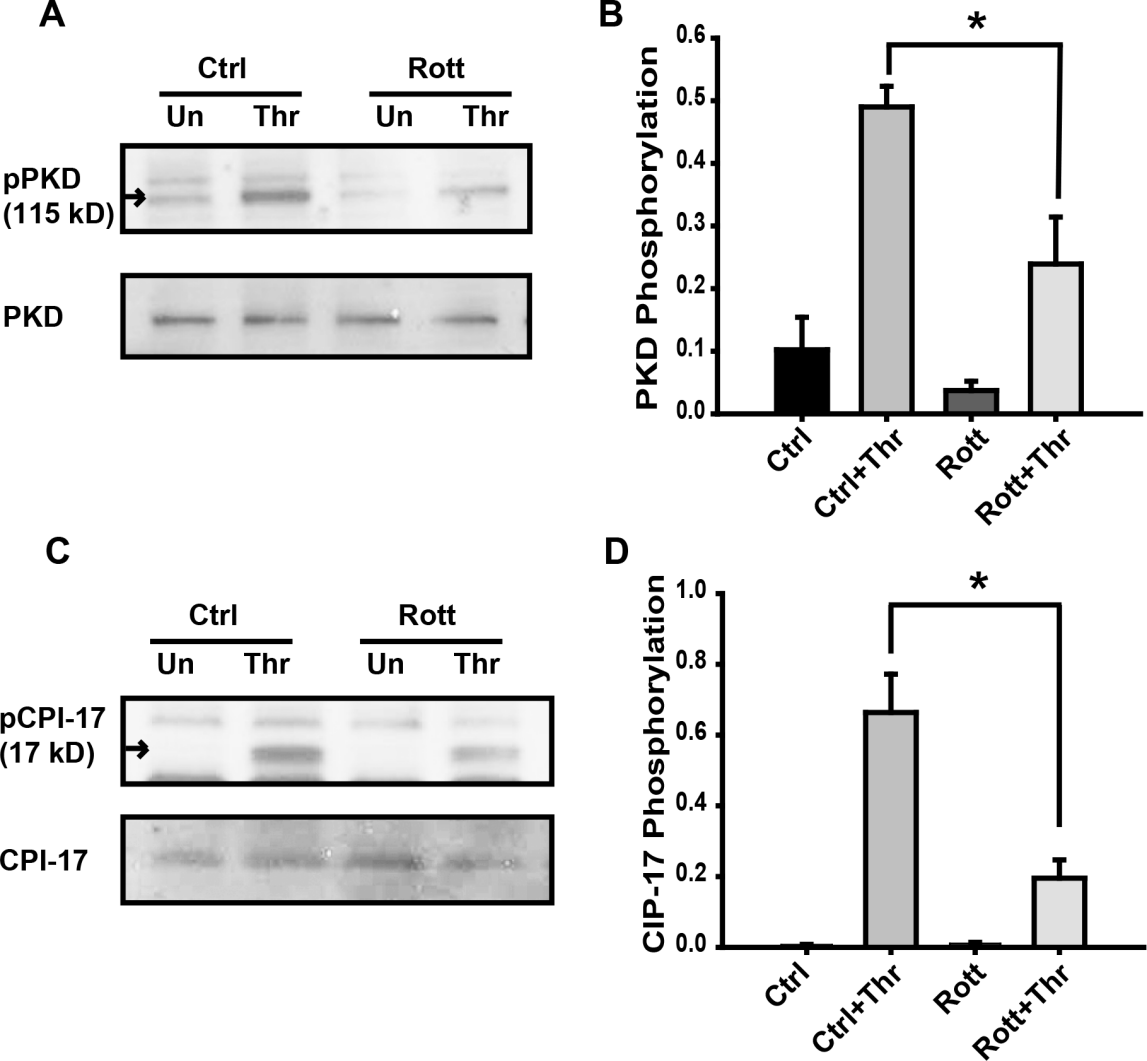
Effect of rottlerin pretreatment on thrombin-induced PKD and CPI-17 phosphorylation. **(A)** EC were pretreated with rottlerin (10 μM, 30 min) followed by stimulation with thrombin (1U/ml, 5 min) and whole cell lysates were immunoblotted with anti-phospho-PKD and anti-PKD antibody. Unstimulated cells (Un) were used as controls. **(B)** Normalized densitometry analysis of PKD phosphorylation is shown (n ≥ 3/condition, * p < 0.05). **(C)** In subsequent experiments using the same conditions, whole cell lysates were immunoblotted with anti-phospho-CPI-17 and anti-CPI-17 antibody. **(D)** Normalized densitometry of CPI-17 phosphorylation is shown. n ≥ 3/condition, * p < 0.05.

PKD (also known as PKCμ;) and CPI-17 are known PKCδ targets involved in cytoskeleton regulation [7, 14, 15, 33]. We examined the effect of PKCδ inhibition on thrombin-induced activation of PKD and CPI-17. As shown in Fig 6A, thrombin induced PKD phosphorylation is partly inhibited in EC pretreated with rottlerin (Fig 6A and 6B). These observations are consistent with the cytosol to membrane translocation of PKCδ shown in Fig 1A. Further, a significant increase of CPI-17 phosphorylation was observed upon thrombin exposure that was once again partially attenuated by rottlerin-pretreatmnet (Fig 6C and 6D).

### PKCδ silencing attenuates thrombin-induced barrier disruption and phosphorylation of MLC, PKD and CPI-17

**Fig 7:**
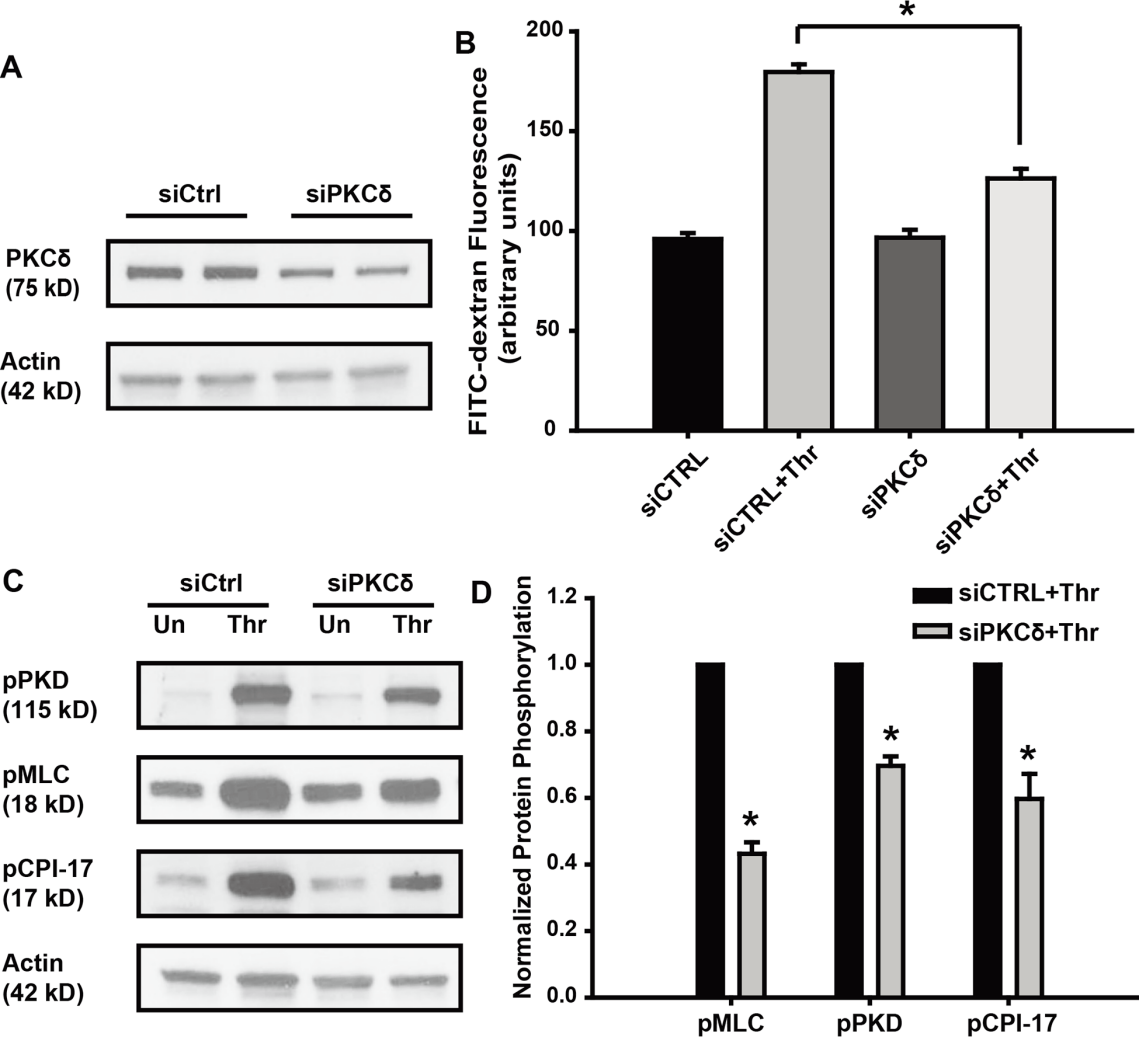
PKCδ silencing attenuates thrombin-induced permeability increase and phosphorylation of MLC, PKD and CPI-17. (**A)** EC were transfected with 100 nM small interfering RNA of PKCδ (siPKCδ) or scramble RNA control (siCTRL). 72 h later whole cell lysates were immunoblotted with antibodies as indicated. (**B)** EC transfected with PKCδ or control siRNA, were treated with thrombin (1 U/ml) for 1 h, and permeability was measured, the plot shows the fluorescence intensity of FITC-dextran (n = 3, * p < 0.05). (**C)** EC transfected with PKCδ or control siRNA were treated with thrombin (1 U/ml) for 10 min, whole cell lysates were immunoblotted with antibodies as indicated. (**D)** Normalized densitometry of MLC, PKD and CPI-17 phosphorylation is shown (n ≥ 3/condition, * p < 0.05).

To address potential limitations in utilizing pharmacologic antagonists or inhibitors [34], specific small interfering RNA were used to reduce expression of the endogenous PKCδ protein, with greater than 50% reduction of PKCδ protein observed 72 h post-transfection (Fig 7A and S1 Fig). Transwell assays were next performed to determine the effect of PKCδ knockdown on the thrombin-induced permeability increase. Compared with scrambled control, knockdown of PKCδ significantly attenuated thrombin-induced barrier disruption (Fig 7B). Decreased thrombin-induced phosphorylation of MLC, PKD and CPI-17 was observed in the siPKCδ group (Fig 7C and 7D), data consistent with the PKCδ inhibitor studies performed.

## Discussion

A breach in the integrity of the lung vasculature can have catastrophic consequences as compromise in the pulmonary EC barrier may result in leakage of fluids and cells into interstitial spaces and precipitate inflammation, cardinal features of syndromes such as acute lung injury and sepsis [35]. Thrombin, a central protease in the coagulation cascade, is an edemagenic factor in the lungs. Moreover, its effects are relevant to wound healing, inflammation and mitogenesis of various cells. Thrombin interacts with its receptor PAR1 on EC, leading to an increase in intracellular calcium, an increase in Rho/ROCK signaling, and inhibition of MYPT1 resulting in increased MLC phosphorylation, actin stress fiber formation, EC contraction, paracellular gap formation, and increased vascular permeability [24]. Using human umbilical vein EC, we have previously shown that thrombin rapidly activates phospholipase D activity that is dependent on a surge of intracellular calcium and PKC activation [36]. In the present study, we observed that PKCδ plays an essential role in thrombin-induced barrier disruption. In thrombin-treated cells, PKCδ is selectively translocated to the membrane compartment from the cytosol, a hallmark of activation of some PKC isoforms. We have also shown here that thrombin-induced PKCδ activation partly promotes RhoA activation and phosphorylation of MLC, PKD and CPI-17, crucial for actin stress fiber formation, cell contraction and vascular leakiness.

PKCδ is a critical regulator of endothelial barrier function and inflammatory responses. In Rats model of ARDS, blocking PKCδ activation with dominate negative peptide would protect animal against inflammation and acute lung injury [37]. Furthermore, studies with PKCδ knock out mice indicated that PKCδ mediated endotoxin-induced lung injury by modulation of inflammatory signaling and pulmonary vascular barrier function [38, 39]. In several epithelial cell and EC models, a role for PKCδ in barrier integrity has been shown. In a monolayer of intestinal Caco-2 cells, hydrogen peroxide induced hyper-permeability was shown to be mediated by the translocation of PKCδ to the membrane fraction which was required for disruption of the microtubule cytoskeleton, whereas the expression of the dominant negative form of PKCδ conferred protection [40]. Also in LLC-PK1 (pig kidney) epithelial cells, over-expression of PKCδ disrupted tight junction permeability [41]. These findings are consistent with a role for PKCδ activation in epithelial cell barrier disruption.

The existence of multiple PKC isozymes has complicated the study of the role of PKC in the regulation of EC barrier integrity [42]. Harrington et al., using rat epididymal microvascular EC, found that overexpression of PKCδ severely blunted thrombin-induced barrier disruption, an effect that could be reversed by rottlerin, a putative inhibitor of PKCδ [9]. In addition, they also observed that PKCδ overexpression significantly enhanced thrombin-induced activity of RhoA that was also suppressed with either rottlerin or upon the expression of a dominant negative form of PKCδ [8]. In contrast, the present study demonstrates that PKCδ is a significant mediator of thrombin-induced EC barrier disruption and the effect can be markedly attenuated by PKCδ inhibition. Notably, our findings are in agreement with a report using rat lung microvascular EC in which PKCδ was shown to promote a loss in EC barrier integrity that could be ameliorated by PKCδ antisense oligonucleotides [7]. These seemingly contradictory results could be explained by the existence of significant micro-heterogeneity in human vascular EC recently reported based on gene expression profiling studies [43]. Another possibility is that the overall effects of PKC are determined by the relative expression of individual PKC isoforms expressed in a particular cell types rather than on any one specific isoform. In a separate study, Bussolino et al. observed that of the three isoforms of PKC expressed in human EC, only PKCα and δ are activated and positively contribute to barrier disruption mediated by platelet-activating factor (PAF) [44]. However, unlike PAF, thrombin had no effect on the translocation of PKCα or β in our study. On the other hand, the study by Harrington et al. using rat epididymal vascular EC suggested that PKCα promotes endothelial barrier dysfunction [9], which was not observed in the present study. Of note, we recognize that there is evidence that rottlerin may not be entirely specific for PKCδ and may target other key intracellular enzymes; however, our studies based on the expression of dominant negative form of PKCδ, which specifically blocking PKC delta activation [45, 46], clearly support our rottlerin studies.

Phosphorylation of MLC plays an important role in cytoskeleton regulation by increasing the mechanochemical interaction between actin and myosin. The levels of phosphorylated MLC are determined by the balance between the activities of MLCK and MLC phosphatase (MLCP) [47]. We previously showed that thrombin-induced EC barrier disruption is highly dependent on MLC phosphorylation mediated by MLCK [48] and PKC activation is required for MLCK-dependent EC barrier responses to thrombin [47]. In coronary venules, ML-7, an MLCK specific inhibitor, significantly attenuates PKC-induced barrier disruption. Here we found that inhibition of PKCδ results in suppression of MLC phosphorylation which suggests that PKCδ could be upstream of MLCK in EC thrombin signaling.

Another possible role for PKCδ in thrombin-induced MLC phosphorylation is MLCP inhibition. MLCP is a heterotrimeric enzyme formed by a 38 kDa phosphatase 1 catalytic subunit (PP1), a 130 kDa targeting subunit (MYPT1) and a 20 kDa subunit with unknown function [49]. CPI-17, a potential substrate for all PKC isoforms [14], selectively inhibits the PP1c subunit of MLCP [50], and we previously showed that CPI-17 is involved in EC cytoskeleton regulation [33]. Here we found that inhibition of PKCδ attenuated CPI-17 phosphorylation suggesting PKCδ may activate MLC by inhibition of MLCP through direct phosphorylation of CPI-17. The other pathway regulating MLCP activity is through MYPT1. ROCK, a key downstream kinase of RhoA GTPase, inhibits MLCP by MYPT1 phosphorylation, ultimately leading to the formation of actin stress fibers [29, 51]. It is therefore possible that the suppressive effect on MLC phosphorylation after PKCδ inhibition we observed is through its inhibitory effect on thrombin-induced RhoA activation.

Rac1 is another well-known small GTPase involved in cytoskeleton regulation known to promote the formation of membrane ruffles and lamellipodia with a concomitant loss in stress fibers [52]. Interestingly, suppression of even basal PKCδ activity with rottlerin resulted in a dramatic increase in Rac1 activation. These results support our finding that activation of PKCδ promotes loss of EC barrier integrity. In addition, a surprising finding is that thrombin appears to have a powerful suppressive effect on Rac1 activation that is independent of PKCδ activity that in turn augments thrombin-induced EC leakiness.

Thrombin-induced cleavage of PAR1 activates G-proteins, G_i_, G_12/13_, and G_q_. G_i_ signaling results in the inactivation of adenylate cyclase, whereas G_12/13_ signaling activates the small GTPase RhoA which ultimately mediates stress fiber formation and focal adhesion reorganization [24]. G_q_ signaling, however, activates phospholipase C, which leads to increases in intracellular calcium and diacylglycerol level. Diacylglycerol then activates PKCδ. Thus in the classical pathway, PKCδ is downstream of G protein signaling that activates RhoA [53]. In the present study, we observed that PKCδ inhibition has a significant suppressive effect on RhoA which suggests that activation of PKCδ has a positive feedback effect on RhoA by an as yet unknown mechanism. In another side, Rac1-GTP was enhanced with Rottlerin, indicated that PKCδ has a suppressive effect on Rac1 activity. It was recently reported that PKD phosphorylates rhotekin leading to RhoA activation by enhancing its membrane association [54]. Given the association of PKD with PKCδ and the fact that PKD is an immediate downstream target of PKCδ [13, 15], PKD signaling may serve as a link between PKCδ and RhoA activation.

**Fig 8:**
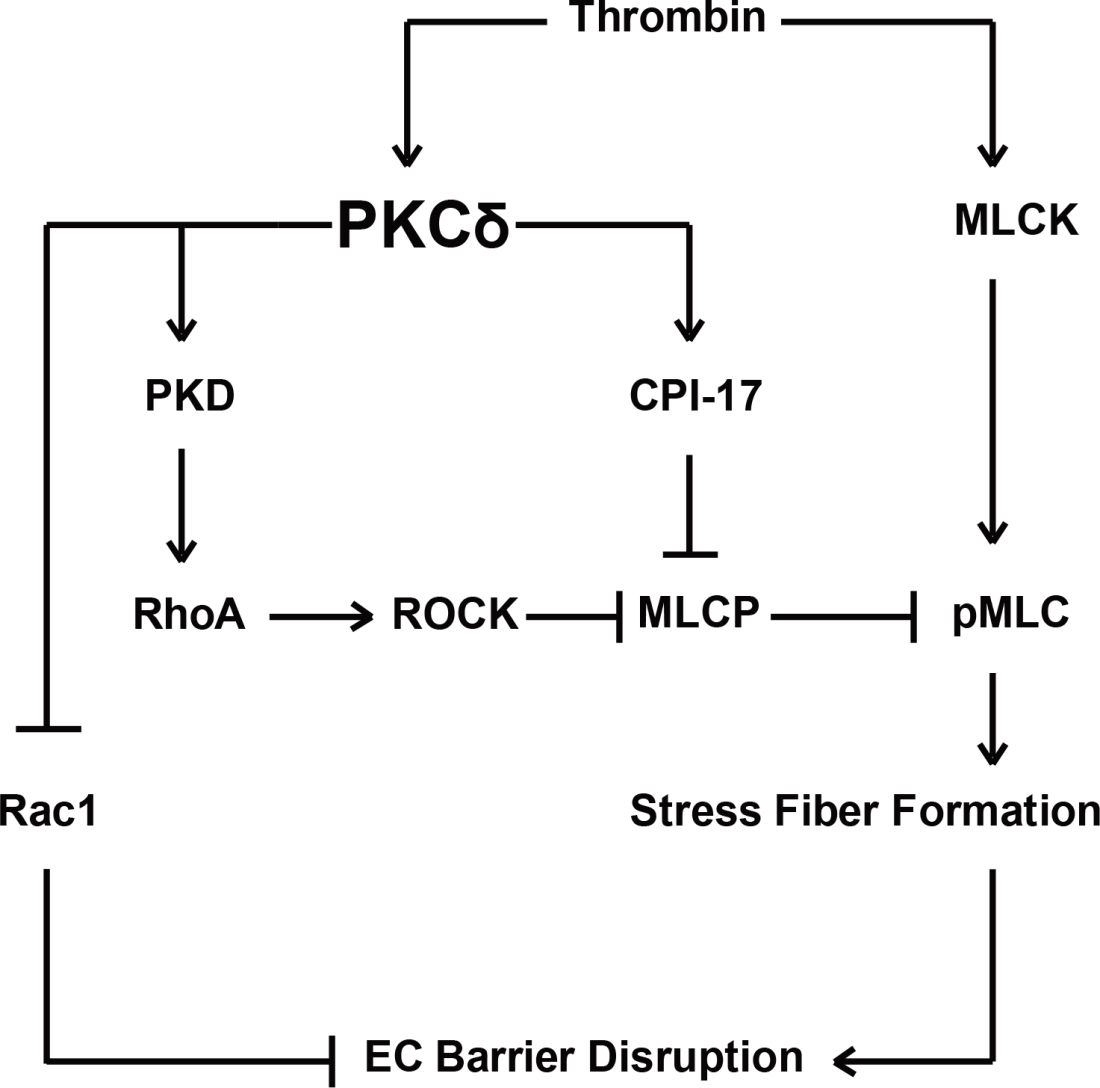
Proposed roles of PKCδ in thrombin-induced human lung EC barrier disruption through a pathway involving MLC and Rho GTPase. Thrombin activates PKCδ that then leads to the activation of PKD and CPI-17. In turn, PKD activates the RhoA pathway inducing MLC activation, while PKCδ phosphorylates CPI-17, a potent MLCP inhibitor, thereby resulting in increased MLC phosphorylation. MLC phosphorylation mediates actin stress fiber formation and contraction. In addition, PKCδ inhibits Rac1 activity blocking its barrier protective effects upon thrombin stimulation.

In conclusion, PKCδ plays a significant role in thrombin-induced loss of human lung EC barrier integrity, findings substantiated by PKCδ inhibitor, studies (rottlerin) expression studies utilizing a dominant negative PKCδ construct and silencing endogenous PKCδ with small interfering RNAs. In addition, we identified PKCδ as upstream of thrombin-induced MLC phosphorylation, RhoA activation and Rac1 inhibition (Fig 8), a confluence of signaling events that mediate stress fiber formation, cell contraction and loss of barrier integrity, while PKD and CPI-17 are potential PKCδ targets involved in this pathway. These studies highlight PKCδ as a therapeutic target in inflammatory lung disorders characterized by the loss of barrier integrity such as acute lung injury and sepsis.

## Supporting Information

S1 Fig PKCδ protein level was reduced by specific siRNA. Densitometry of individual Bands was quantified and normalized. siCtrl: control; siPKCδ: transfected with PKCδ specific siRNA (n > 3).
(TIF)
