# Supplementary figures and images for: Regulation of Thrombin-Induced Lung Endothelial Cell Barrier Disruption by Protein Kinase C Delta
Source: PLoS One. 2016 Jul 21;11(7):e0158865. doi: 10.1371/journal.pone.0158865 (PMC4956111; doi:10.1371/journal.pone.0158865)

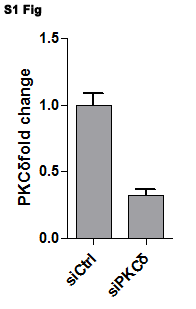

Supplement: S1 Fig — Densitometry of individual Bands was quantified and normalized. siCtrl: control; siPKCδ: transfected with PKCδ specific siRNA (n > 3). (TIF) [file pone.0158865.s001.tif]
